# Steroid-Responsive Gradenigo’s Syndrome Mimicking Subdural Hematoma

**DOI:** 10.7759/cureus.19547

**Published:** 2021-11-13

**Authors:** Yi Liu, Po-Kuan Yeh, Yu-Pang Lin, Yueh-Feng Sung

**Affiliations:** 1 Department of Neurology, Tri-Service General Hospital, National Defense Medical Center, Taipei, TWN; 2 Department of Radiology, Tri-Service General Hospital, National Defense Medical Center, Taipei, TWN

**Keywords:** gradenigo syndrome, gradenigo's syndrome, sixth nerve palsy, retro-orbital pain, otorrhea, subdural hematoma, petrous apicitis

## Abstract

Gradenigo's syndrome (GS) is featured by a clinical triad of otorrhea, retro-orbital pain, and a sixth nerve palsy. Clinical examination is crucial prior to considering neuroimaging. The majority of cases are secondary to infection thus requiring long-term broad-spectrum antibiotics; severe cases also require surgical intervention for risk of intracranial abscess or even death.

The patient was a 35-year-old female who presented with right temporal headache and right retro-orbital pain. The initial diagnosis from the local clinic was of subdural hemorrhage. Cranial nerve (CN) VI paresis was noted upon examination and inflammatory process was documented based on brain MR. The patient was diagnosed with Gradenigo's syndrome and administered antibiotics and steroids. Symptoms recurred after cessation of steroids and once antibiotics-related fever developed. The symptoms resolved after stopping the antibiotics and reintroducing steroids. The MRI performed after three months recorded no brain inflammation.

We report a Gradenigo's syndrome caused by chronic inflammation with good response to steroids. To our best knowledge, there were merely approximately 80 patients who were reported with Gradnigo or Gradenigo’s syndrome before. Infection comprised 76% of cases, thus broad-spectrum and long-term antibiotics use have been emphasized instead of steroid use. However, steroids also play an important role in reducing nerve injury by edematous change.

## Introduction

Gradenigo’s syndrome (GS) was first described in a case series by Giuseppe Gradenigo in 1904 [1]. GS is characterized by the clinical triad of otorrhea, retro-orbital pain, and sixth nerve palsy. Moreover, it is commonly caused by the progression of untreated or incompletely treated otitis media. However, the classical triad may not always be observed in GS. Even in the original series by Gradenigo, only 42% (24 of the 57 cases) of patients presented with the classic triad. Other patients exhibited the complete triad of symptoms without evidence of petrous bone inflammation.

The boundaries of the petrous apex include several neurological structures, such as the Dorello canal near the medial superior tip of the petrous apicitis (PA) through which the abducens nerve passes, and the cisternal trigeminal nerve, which can be affected by inflammation. Deficits in cranial nerves (CNs) II-X, most commonly CN VI, can occur if the infectious process extends to the skull base or cavernous sinus [2].

The diagnostic procedures include a comprehensive history taking, particularly in patients with preceding purulent otalgia, including if there was trauma, fever, cranial nerve dysfunction, decreased hearing, vertigo, tinnitus and activities that can affect the canal or tympanic membrane. Contrast-enhanced brain computed tomography (CT) scan and magnetic resonance imaging (MRI) must be performed to identify the extent of erosion in bony structures and soft tissue involvement if there is evidence of meningeal and brain parenchyma invasion. Other additional procedures include bone scan, lumbar puncture, pure-tone Audiometry (PTA), and fundoscopy. Broad-spectrum antibiotics are administered then downgraded according to sensitivity tests. In more severe cases, refined surgical techniques were used, and the incidence of intracranial complications decreased to 0.04%-0.15% [3]. The complications of cranial base osteomyelitis and petrous apicitis may include labyrinthitis, meningitis, intracranial abscess, retropharyngeal, abscess, venous sinus thrombosis and cranial neuropathies.

## Case presentation

A 35-year-old woman without a significant medical history presented to a regional hospital due to severe pain over the right temporal and right retro-orbital area for two weeks, followed by double vision and right facial numbness for three days. The patient claimed the absence of fever, ear pain, hearing loss, and head trauma. However, she had a habit of picking the right ear for several years. Non-contrast-enhanced brain CT scan showed a high-density region along the right falx cerebri to the tentorium cerebelli. Thus, she was referred to our hospital for suspected subdural hemorrhage.

**Figure 1:**
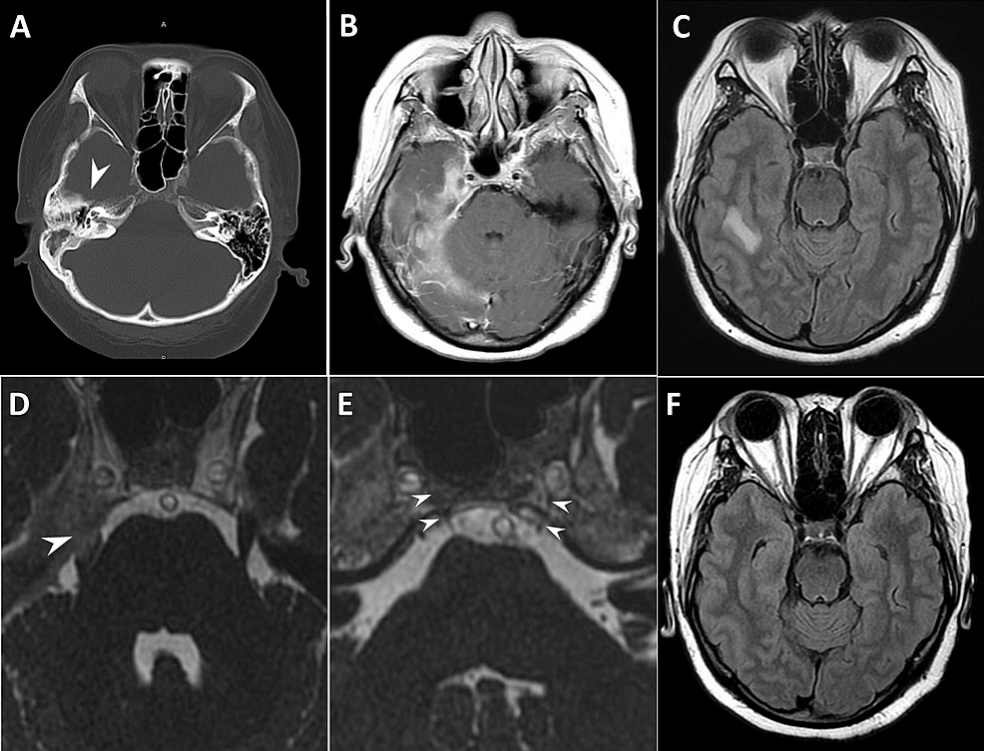
Brain computed tomography (CT) scan and magnetic resonance imaging (MRI) results indicated Gradenigo’s syndrome, which was characterized by pachymeningitis, leptomeningitis, and cerebritis. Non-contrast-enhanced brain CT scan showed right petrous apicitis with ill-defined irregular edges, and there was soft tissue in the mastoid cavity and less pneumatized mastoid air cell system (arrowhead) as compared to the opposite side suggesting chronic otitis media. (A). Brain MRI revealed pachymeningitis, leptomeningitis, and cerebritis involving the right tentorium cerebelli (B) and the right temporal region (C). Moreover, there was inflammation in the Meckel cave and CN V (arrowhead) (D). The right Dorello canal was swollen compared with the left one (arrowheads) (E). Follow-up MRI revealed the complete resolution of previous signal abnormalities. Cerebritis in the right temporal area [(F) vs (C)].

**Figure 2:**
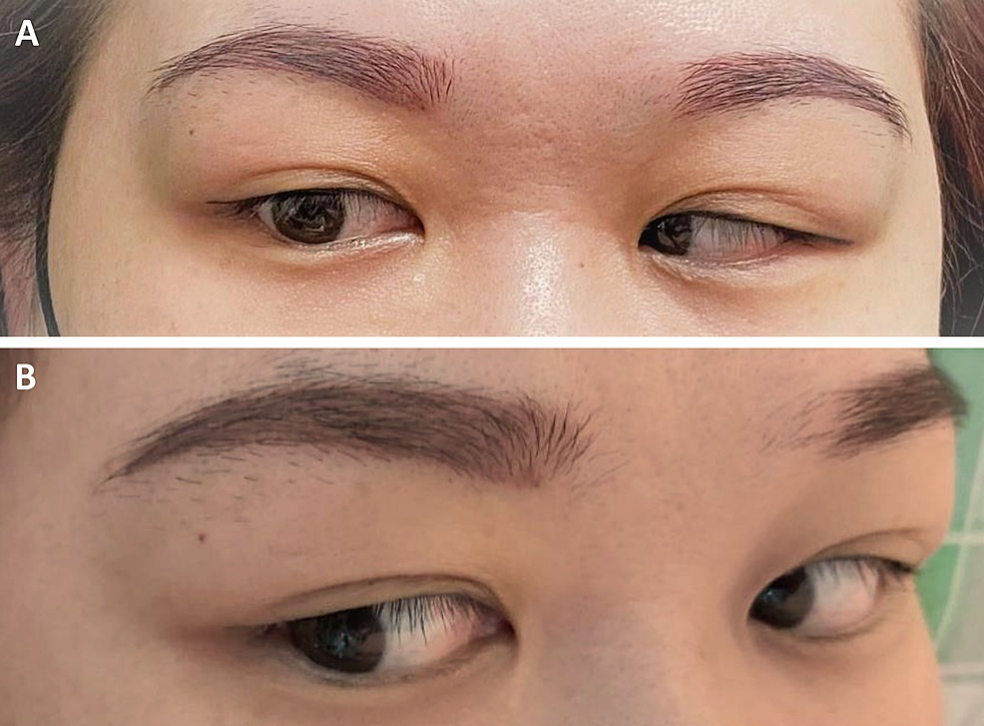
Right abducens nerve paresis improved after steroid treatment. (A) At the initial presentation, right abducens nerve paresis was observed, and the patient presented with diplopia, particularly looking toward the right side. (B) Diplopia disappeared gradually after 5 days of steroid treatment, and there were no limitations in eyes movement.

Neurological examination revealed right abducens palsy and paresthesia in the area involving the maxillary and mandibular branches of the right trigeminal nerve. The tympanic membrane was normal. Blood examinations showed a normal white blood cell count (6.16 × 103/uL), but elevated inflammatory marker levels (erythrocyte sedimentation rate [ESR]: 86 mm/h, C-reactive protein level: 1.81 mg/dL). The cerebrospinal fluid assessment results were normal, except for elevated total protein levels (88 mg/dL), and cultures were sterile. Moreover, autoimmune and coagulation disorders including HIV were ruled out. The homocysteine, antinuclear antibodies (ANA), rheumatic factor, antistreptolysin O titer (ASOT), C3, C4, IgG, IgM, IgA, d-dimer, anti-thrombin III, lupus anticoagulant, protein C and S, rapid plasma reagin (RPR), anti-cardiolipin IgG, alpha-fetoprotein (AFP), carcinoembryonic antigen (CEA), cancer antigen (CA)-125, CA-153, CA19-9, squamous cell carcinoma (SCC), and hemoglobin A1C (HbA1c) levels and thyroid function were normal. After adjusting the window of initial brain CT at clinic, there was less pneumatized mastoid air cell system suggesting chronic otitis media at right ear (Figure 1A). Contrast-enhanced brain MRI showed pachymeningitis involving the right tentorium cerebelli and the right temporal region, leptomeningitis in the right temporo-occipital region (Figure 1B), cerebritis in the right temporal lobe (Figure 1C), inflammation in the right side of the Meckel cave (Figure 1D) and the Dorello canal (Figure 1E), and right otomastoiditis with petrous apicitis. Thus, the patient was diagnosed with GS, and broad-spectrum antibiotics were administered. Symptoms resolved five days after steroid treatment (Figure 2). However, the patient developed high fever after two weeks, which was refractory to antipyretic medications. Next, she presented with generalized skin rash, but not until developing a drug reaction with eosinophilia and systemic symptoms (DRESS) for lacking of eosinophilia nor internal organs dysfunction [4]. Her symptoms initially improved after methylprednisolone therapy but recurred after discontinuation. To rule out infection, right modified radical mastoidectomy and myringotomy with Grommet insertion were performed for pathologic assessment. Results showed chronic inflammation with fibrosis. Since the bacterial culture results were negative, antibiotic therapy was discontinued, and methylprednisolone therapy was maintained. Fever then subsided after two days, and symptoms improved simultaneously. The patient was discharged after one month, with complete resolution of symptoms and without neurological sequelae. Although the patient was asymptomatic, she was treated with methylprednisolone 4 mg 0.5-2 tabs per day due to elevated ESR levels in the outpatient department. Follow-up brain MRI was performed after three months, and results showed complete resolution of signal abnormalities (Figures 1C, 1F).

## Discussion

Herein, we describe a young woman with idiopathic GS that responded well to steroids. Data that can be used as a guide by clinicians in the workup, treatment, and assessment of similar cases is limited. Hence, more scientific evidence must be collected.

We searched Medline, PubMed, and Google scholar and Google search engines using the following keywords: (Gradnigo OR Gradenigo’s syndrome OR Gradenigo’s OR apicitis OR idiopathic Gradnigo). Then, the references and citations of each study, published from 1980 to 2021, were assessed. Reports describing apicitis but not GS were excluded. Finally, we retrieved 60 relevant articles, which included 80 patients diagnosed with GS (Table 1) [1,5-63]

**Table 1:** Previous published cases of Gradnego's syndrome from 1980 to 2021/06 * Pseudomonas aeruginosa, gram-positive cocci in pair, Proteus mirabilis, Alcaligenis faecalis, Enterococcus faecalis, Citrobacter koseri, and Bordetella trematum, pseudomonas aeruginosa, staphylococcus aureus, Streptococcus pneumonia, Klebsiella pneumonia Abx: antibiotics AOM: acute otitis media CSOM: chronic suppurative otitis media NA: not available NPC: nasopharyngeal carcinoma Surgery: radical mastoidectomy with petrous apicectomy, myringotomy TB: tuberculosis

Reference	Age (yrs)/gender	Medical history/Preceding events	Etiology	Treatment	Prognosis
1980, Paolucci, et al. [5]	NA	NA	Metastasis of prostatic carcinoma	NA	NA
1983, Chole, et al. [6]	78M; 57M; 18F; 46M; 46M; 73M; 65M; 28M	Histiocytic lymphoma; cholesteatoma; severe deep ear pain; otorrhea and hearing loss*; otorrhea(12yrs); healthy; deafness; congenital petrous apex cholesteatoma	Bacterial infection	All surgery	Vernet's syndrome and died (73M)
1984, Capanna, et al. [7]	19M	NA	Gunshot	craniotomy	NA
1988, Ggraaf, et al. [8]	58F	Otosclerosis, right stapes	Bacterial infection	NA	NA
1989, Norwood, et al. [9]	13M	Healthy	T Cell lymphoma	chemotherapy and radiotherapy	NA
1991, Grewal, et al. [10]	3 Patients	NA	TB	NA	NA
1992, Hehl, et al. [11]	36F_bilateral	NA	NA	hyperbaric oxygenation	NA
1993, Linstrom, et al. [12]	42M	HIV	B-cell non-Hodgkin's lymphoma	chemotherapy	NA
1995, Hardjasudarma, et al. [13]	32M	Healthy	Bacterial infection	Abx	NA
1997, Morales, et al. [14]	44M	HIV, right ear surgery recent	Bacterial infection	Abx	NA
1998, R Bourne, et al. [15]	45M	myeloma	Intracranial plasmacytoma	NA	NA
1999, Minotti, et al. [16]	47F; 36F(bilateral)	Healthy; bilateral chronic otitis media	Bacterial infection	Surgery and Abx; Surgery, Abx, dexamethasone, mannitol, and Dilantin.	NA
2000, M Motamed, et al. [17]	78M	T2DM	Bacterial infection	Abx	Vernet’s syndrome, aspiration pneumonia.
2001, Penas-Prado M, et al. [18]	53M	Healthy	NPC	Radiotherapy	NA
2002, L Mathew, et al. [19]	25M; 12M	Healthy; Healthy	Bacterial infection	Abx, mastoidectomy; Abx, mastoidectomy	NA
2004, Sherman, et al. [20]	55M	T2DM	Bacterial infection	6wks Abx(ceftri), myringotomy	NA
2005, Burston, et al. [21]	6M(bila.), 71M	Nil, bilateral chronic suppurative otitis media, and a left pars tensa perforation	Bacterial infection	Abx(cef), myringotomies; Abx(metro 4w, ceftri 6w, Cipro 6w, clinda)Streptococcus milleri	remained well over the ensuing 12 months
BradleyJC, [22]	47M	Tobacco abuse and hepatitis B and C	NPC	Chemotherapy+ RT	NA
2006, Jana, et al. [23]	15M	NM	NPC	NM	NM
2007, Bravo, et al. [24]	53M	Healthy	Bacterial infection	chloramphenicol and ceftriaxone, for 21 days	NA
2010, Ilias Kantas, et al. [25]	24F	infection of the upper respiratory tract one month ago	Bacterial infection	Abx Streptococcus pneumoniae	hearing loss was recovered
2010 Tornabene, et al. [26]	60F	breast cancer	Bacterial infection	Abx	complete resolution of her facial pain and right abducens nerve palsy after 2 months
2011 José Luiz Pedroso, et al. [1]	33F	smoked for 9 years.	Diffuse giant B-cell non- Hodgkin’s lymphoma and a nasopharyngeal mass	chemotherapy	NA
2012 Burak Ulkumen, et al. [27]	56M	Healthy	Bacterial infection	Abx	NA
2012 Delgado, et al. [28]	28F	Healthy	Bacterial infection	Staphylococcus aureus	NA
2012, Esteban Espínola Duarte, et al. [29]	29M	deaf-mute	NPC	Chemotherapy+ RT	NA
2013, Bhatt, et al. [30]	72M	CSOM	Aspergillus	prednisolone 60 mg QD+ Augmentin, ceftri, metro, liposomal amphotericin B, voriconazole	facial palsy was still present at three months’ follow up and was managed with tarsorrhaphy.
2013, Macasaet, et al. [31]	54F	Ear discharge 6 months prior	post-mastoidectomy recurrent chronic suppurative otitis media with cholesteatoma formation	Abx(ceftri) mastoidectomy with translabyrinthine	hoarseness and lateral gaze palsy remained.
2014, Chen, et al. [32]	64F, 33F, 58M, 45M	Pulmonary TB; COM; HTN; Healthy; previous tympanoplasty	TB	Mastoidectomy;Abx for 13 months ; mastoidectomy, Abx for 12 months; mastoidectomy, Abx; mastoidectom, Abx	Recovery of CN deficits after operation 20 d to 4 months
2014, Khalatba ri, et al. [33]	46M	NA	Solitary Osseous plasmacytosis	Radiotherapy	No recurrence or progression to multiple myeloma 4 yrs later
2014, Valles, et al. [34]	36F	NA, 23 weeks pregnant	Sinus thrombosis	Enoxaparin	NA
2014, Yuvatiya Plodpai, et al. [35]	63M	NA	Bacterial infection	Ceftazidime + levofloxacin	Complete recovery 2 months later
2015, Lattanzi, et al. [36]	60F	NA	Cholesterol granuloma.	NA	NA
2016, Elham Ouspid, et al. [37]	65F	NA	NPC	Radiotherapy	4 months later patient expired due to fulminant sepsis
2017, Jbali Souheil, et al. [38]	55F	Budd Chiari syndrome,	Sphenoid sinus tumor	Radiotherapy	NA
2016, Jensen, et al. [39]	5M, 46F, 70F, 13M	AOM 1 mo, AOM 1 mo	Bacterial infection complicate with sinus thrombosis,; Hemolytic streptococcus group A; Streptococci species; GNB, Candida	Mastoidectomy, Abx, LMWH; Abx; Abx, mastoidectomy; Abx, surgery	no relapse in 18 months, nil, nil, ni;
2016, Nayya, et al. [40]	55F	NA	Bacterial infection	Abx, mastoidectomy	well
2017, Grade, et al. [41]	40 years follow up of 44 patients	Mainly infection	Abx and surgery	1 of them died
2017, H Verma, et al. [42]	30F	left ear discharge since 10-15 years	congenital neuroenteric cyst and bacterial infection	Abx	NM
2017, Jensen, et al. [43]	9F	year-long history of CSOM	Bacterial infection	Abx and mastoidectomy	mortality
2017, Nicholas Taklalsingh, et al. [44]	58M	one-year history of CSOM	Bacterial infection	Abx and mastoidectomy	Residual neurological sequalae
2017, Suresh Mani, et al. [45]	25M	NA	Bacterial infection*	Abx at least	NA
2017, Tayebeh Kazemi, et al. [46]	33M	NA	Bacterial infection	Abx	temporal bone CT 6 weeks later iomproved
2018, Ahmad, et al. [47]	61M	NA	Bacterial infection	Abx and exploration of mastoid and middle ear.	successful result after postoperative follow-up
2018, Aina Brunet-Garcia, et al. [48]	40M	NM	Bacterial infection	Abx and surgery	NM
2018, Asude Aksoy, et al. [49]	52M	Healthy	NPC	Abx and steroids, CCRT	without any medical treatment and complaint in follow-up
2018, Rajneesh Thaku, et al. [50]	51M	on and off discharge from his left ear since the age of 25 years	Bacterial infection	Abx, mastoidectomy and petrous exploration	Loss follow up
2019, Petrenko, et al. [51]	22F	T1DM	Mucormycosis	Amphotericin B	NA
2019, Conor Bowman, et al. [52]	67M	NA	Bacterial infection	Abx	has not been readmitted
2019, Esmanhotto, et al. [53]	37F	SLE	Bacterial infection	Abx	NA
2019, Savasta, et al. [54]	11M	recurrent upper airways infections, frequently resulting in episodes of AOM since age 4	Bacterial infection	Abx and steroids	symptomsfree for the 30 months follow-up
2019, Rossi, et al. [55]	4F	Recent sinusitis	Bacterial infection	Abx and surgery	notable improvement after 2 week
2020, Chandran, et al. [56]	54F; 23F	3-year otalgic disease; contact with TB	TB	Anti-TB therapy	NA
2020, Guilherme Correa Guimaraes, et al. [57]	63F	HTN, T2DM	Bacterial infection*	Abx, enoxaparin	Complicated with cavernous sinus Thrombosis; total recovery 4 months after the first symptom presentation
2020, Hodges, et al. [58]	24M	Asthma	Bacterial infection, cholesterol granuloma	Surgery, steroids, Abx	NA
2020, Mclaren, et al. [59]	5F	Healthy	Bacterial infection	Abx	Symptoms free
2020, Meena V. Kale, et al. [60]	3male 30-40yrs	NA	Bacterial infection*	Abx up to 8wks	NA
2020, Nilam, et al. [61]	57M	Previous ear infection	Bacterial infection and chronic inflammation	Mastoidectomy, Abx	lateral rectus palsy completely recovered
2020, Ghammam, et al. [62]	6F	Healthy	Bacterial infection	Abx	full recovery
2021, Parekh, et al. [63]	71M	T2DM	NA	surgical and medical management	Vernet's syndrome, died a few weeks later

Male predominance was observed, with a male-to-female ratio of 1.74. The average age of patients is 41.9, with the youngest aged four and the oldest 78. Approximately 76% of patients had infection. Six patients presented with nasopharyngeal carcinoma, five with cholesterol granuloma, and three with lymphoma. Moreover, other conditions including primary tumor, plasmacytoma, sinus thrombosis, and gunshot were reported. It’s crucial to identify head trauma for relatively high frequency of traumatic pathology in otolaryngologic practice, especially in young adults and males [64]. Immunosuppressed patients (HIV, type 2 diabetes mellitus, etc) were considered risk factors. Further, preceding otologic surgery and infection were commonly observed in all cases (Table 2).

**Table 2:** Epidemiology and causes of Gradenigo's syndrome Male is predominant with male to female ratio 1.74, average age is 41.9 with the youngest one aged 4 and the oldest aged 78. Infection composed of 76%; there were 12 cases of malignance, 5 cases of cholesterol granuloma, and also sinus thrombosis and gunshot. Immunocompromises like HIV (case number=2) and type 2 diabetes mellitus (case number=4) were considered as the risk factors, also, preceding otologic surgery and infection were found common in all reported cases.

Classification (available cases/All cases)	Detailed
Gender (74/80)	M:F=1.74:1 (47M, 27F)
Age (74/80)	Average 41.9 years old
Etiology (79/80)	Infection (n=60); including bacterial infection (n=49), tuberculosis (n=9), aspergillus (n=1), and mucormycosis (n=1)
	Malignance (n=12); including nasopharyngeal carcinoma (n=6), metastasis of prostatic carcinoma (n=1), mass (n=1), lymphoma (n=3) and plasmacytoma (n=1)
	Cholesterol granuloma (n=5)
	Others (n=2); including gunshot (n=1) and sinus thrombosis (n=1)

To the best of our knowledge, this is the first case of chronic inflammatory GS. Although the habit of ear picking does not cause otitis media but may cause otitis externa, clinicians should raise the concern of malignant otitis externa and skull base osteomyelitis caused by ear picking, particularly in immunocompromised patients. In cases of infection, high-dose broad-spectrum antibiotics are recommended. Moreover, surgery is indicated in more severe cases. The possible complications of GS include labyrinthitis, meningitis, intracranial abscess, venous sinus thrombosis, and carotid artery stenosis.

The current case is clinically defined as classic GS, which is attributed to chronic inflammation. In our patient, it was difficult to differentiate hemorrhage from inflammation due to focal high-density dural thickening on non-contrast-enhanced brain CT scan. Hence, the condition was initially misdiagnosed in the regional hospital. Subdural hemorrhage in young women is rare, and emergent vascular events including venous thrombosis must be ruled out as first priority for the opposite management manner and major complication if left untreated. Based on both clinical characteristics and brain MRI findings, hemorrhage and thrombosis were ruled out, and the patient was diagnosed with GS. Furthermore, we considered chronic inflammation correlated with GS after ruling out bacterial (tuberculous), fungal, and viral infections. In addition, there is no evidence of venous thrombosis or malignancy based on the assessments performed using the current diagnostic tools. We believe that the pathophysiology was associated with chronic inflammation; hence, the patient substantially benefited from steroid treatment.

Brain radiography revealed petrous apicitis that developed into leptomeningitis, pachymeningitis, and cerebritis. Even without evidence of bacterial infection, meropenem, vancomycin, and metronidazole were still administered initially via intravenous infusion to prevent progression into brain abscess and, subsequently, other morbidities and even mortality.

Steroid is used for the treatment of active inflammation, and it may be beneficial for cases with nerve compression. In our case, symptoms significantly improved after one dose of methylprednisolone 500 mg STAT. Then, methylprednisolone 8 mg every eight hours for three days was administered based on the study of Kazemi et al. [46]. However, in the current case, the condition was associated with idiopathic inflammatory process rather than pathogenic infection. Thus, prolonged treatment with steroids, rather than antibiotics, might be more suitable. We extended the treatment with methylprednisolone 8 mg every eight hours for six days, followed by a dose of 8 mg every 12 hours. Then, the treatment was changed to oral methylprednisolone 4 mg every 12 hours for three days and then once daily.

In a previous case, a teenager was finally diagnosed with Tolosa-Hunt syndrome with an apparent presentation of GS [2]. In another case, mastoiditis complicated by GS was attributed to immune-induced hypertrophic pachymeningitis [3]. There is no evidence confirming the efficacy of steroid, even though it has beneficial effects against inflammation and nerve damage. In several cases of GS, combination treatment with antibiotics and steroids has been effective [34,46,54].

Therefore, cautious clinical history taking, physical examination, and neuroimaging are required to diagnose GS. In most cases, the condition is caused by bacterial infections requiring broad-spectrum antibiotics. However, patients with chronic inflammation can have similar presentations and imaging findings and may require steroid treatment. Also, in the present case which developed also drug-related reactions, a complete allergy panel should be performed to exclude cross reactivity and exposure to environmental factors increasing chronic inflammation [65].

## Conclusions

This is the first case of GS due to chronic mastoiditis (asymptomatic) based on radiological features; patients might miss subtle aura symptoms like past otalgia, aural fullness, dull pain etc.; which successfully resolved after steroid treatment. Unlike in previous cases in which patients were primarily treated with antibiotics, the current study highlighted the importance of steroids in treating inflammation and reducing nerve edema in GS after the management of infection.

## References

[1] Pedroso JL, de Aquino CC, Abrahão A (2011). Gradenigo's syndrome: beyond the classical triad of diplopia, facial pain and otorrhea. Case Rep Neurol.

[2] Reddy RK, Reddy RK, Jyung RW, Eloy JA, Liu JK (2016). Gruber, Gradenigo, Dorello, and Vail: key personalities in the historical evolution and modern-day understanding of Dorello's canal. J Neurosurg.

[3] Hafidh MA, Keogh I, Walsh RM, Walsh M, Rawluk D (2006). Otogenic intracranial complications. a 7-year retrospective review. Am J Otolaryngol.

[4] Vrinceanu D, Dumitru M, Stefan A, Neagos A, Musat G, Nica EA (2020). Severe DRESS syndrome after carbamazepine intake in a case with multiple addictions: a case report. Exp Ther Med.

[5] (2021). Metastatic syndrome involving multiple cranial nerves on the right. https://pubmed.ncbi.nlm.nih.gov/7466206/.

[6] Chole RA, Donald PJ (1983). Petrous apicitis. Clinical considerations. Ann Otol Rhinol Laryngol.

[7] Capanna AH (1984). Traumatic intracranial aneurysm and Gradenigo’s syndrome secondary to gunshot wound. Surg Neurol.

[8] deGraaf J, Cats H, deJager AE (1988). Gradenigo’s syndrome: a rare complication of otitis media. Clin Neurol Neurosurg.

[9] Norwood VF, Haller JS (1989). Gradenigo syndrome as presenting sign of T-cell lymphoma. Pediatr Neurol.

[10] Grewa lD, Baser B, Shahani RN (1991). Tuberculous otitis media presenting as complications: report of 18 cases. Auris Nasus Larynx.

[11] Jackler RK, Parker DA (1992). Radiographic differential diagnosis of petrous apex lesions. Am J Otol.

[12] Linstrom CJ, Pincus RL, Leavitt EB, Urbina MC (1993). Otologic neurotologic manifestations of HIV-related disease. Otolaryngol Head Neck Surg.

[13] Hardjasudarma M, Edwards RL, Ganley JP (1995). Magnetic resonance imaging features of Gradenigo’s syndrome. Am J Otolaryngol Neck Med Surg.

[14] Morales C, Tachauer A (1997). Gradenigo syndrome in a human immunodeficiency virus-positive patient. Arch Intern Med.

[15] Bourne RR, Maclaren RE (1998). Intracranial plasmacytoma masquerading as Gradenigo's syndrome. Br J Ophthalmol.

[16] Minotti AM, Kountakis SE (1999). Management of abducens palsy in patients with petrositis. Ann Otol Rhinol Laryngol.

[17] Motamed M, Kalan A (2000). Gradenigo's syndrome. Postgrad Med J.

[18] Penas-Prado M (2001). Gradenigo syndrome as the form of presentation of nasopharyngeal carcinoma. Rev Neurol.

[19] Mathew L, Singh S, Rejee R, Varghese AM (2002). Gradenigo’s syndrome: findings on computed tomography and magnetic resonance imaging. J Postgrad Med.

[20] Sherman SC, Buchanan A (2004). Gradenigo syndrome: a case report and review of a rare complication of otitis media. J Emerg Med.

[21] Burston BJ, Pretorius PM, Ramsden JD (2005). Gradenigo's syndrome: successful conservative treatment in adult and paediatric patients. J Laryngol Otol.

[22] Nasopharyngeal Carcinoma Presenting as Gradenigo’s Syndrome. https://www.ttuhsc.edu/medicine/ophthalmology/documents/gradenigomanuscript.pdf.

[23] Jana AK, Jaswal A, Sikder B, Jana U, Nandi TK (2006). Nasopharyngeal carcinoma presenting as Gradenigo's syndrome. Indian J Otolaryngol Head Neck Surg.

[24] Bravo D, Machová H, Hahn A (2007). Mastoiditis complicated with Gradenigo syndrome and a hypertrophic pachymeningitis with consequent communicating hydrocephalus. Acta Otolaryngol.

[25] Kantas I, Papadopoulou A, Balatsouras DG, Aspris A, Marangos N (2010). Therapeutic approach to Gradenigo's syndrome: a case report. J Med Case Rep.

[26] Tornabene S, Vilke GM (2010). Gradenigo's syndrome. J Emerg Med.

[27] Ulkumen A, Kaplan Y (2012). Conservative treatment of Gradenigo’s syndrome triggered by acute otitis media. Pak J Med Sci.

[28] Delgado ME, Del Brutto OH (2012). Teaching neuroimages: Gradenigo syndrome. Neurology.

[29] Duarte E, Roig J, Arias J, Amarilla P, Ferreira A, Ortiz H (2012). Gradenigo syndrome in nasopharyngeal carcinoma. Int Arch Otorhinolaryngol.

[30] Bhatt YM, Pahade N, Nair B (2013). Aspergillus petrous apicitis associated with cerebral and peritubular abscesses in an immunocompetent man. J Laryngol Otol.

[31] Macasaet MA, Cruz ET (2018). Vocal cord paralysis and dysphagia as sequelae of Gradenigo syndrome. Philipp J Otolaryngol Neck Surg.

[32] Chen PY, Wu CC, Yang TL, Hsu CJ, Lin YT, Lin KN (2014). Gradenigo syndrome caused by nontuberculous mycobacteria. Audiol Neurootol.

[33] Khalatbari MR, Hamidi M, Moharamzad Y (2014). Gradenigo’s syndrome as first presentation of solitary osseous plasmacytoma of the petrous apex. Arch Iran Med.

[34] Valles JM, Fekete R (2014). Gradenigo syndrome: unusual consequence of otitis media. Case Rep Neurol.

[35] Plodpai Y, Hirunpat S, Kiddee W (2014). Gradenigo's syndrome secondary to chronic otitis media on a background of previous radical mastoidectomy: a case report. J Med Case Rep.

[36] Lattanzi S, Cagnetti C, Di Bella P, Provinciali L (2015). Mystery case: cholesterol granuloma of the petrous apex in Gradenigo syndrome. Neurology.

[37] Ouspid E, Sariaslani P (2016). Gradenigo syndrome as the form of presentation of nasopharyngeal. Zahedan J Res Med Sci.

[38] Souheil J, Mohamed D, Sawssen D (2017). Gradenigo syndrome and primitive sphenoid sinus cancer. Egypt J Ear, Nose, Throat Allied Sci.

[39] Jensen PV, Hansen MS, Møller MN, Saunte JP (2016). The forgotten syndrome? Four cases of Gradenigo's syndrome and a review of the literature. Strabismus.

[40] Nayyar SS, Gupta AK (2016). Gradenigo’s syndrome and petrous apicitis: a rare complication of com: case report. J Evol Med Dent Sci.

[41] Gadre AK, Chole RA (2018). The changing face of petrous apicitis-a 40-year experience. Laryngoscope.

[42] (2021). Post-Tympanoplasty Gradenigo Syndrome - Don’t Panic. https://www.researchgate.net/publication/316685379_Post-tympanoplasty_Gradenigo_Syndrome_-_Don.

[43] Jensen PV, Avnstorp MB, Dzongodza T, Chidziva C, von Buchwald C (2017). A fatal case of Gradenigo's syndrome in Zimbabwe and the Danish-Zimbabwean ENT collaboration. Int J Pediatr Otorhinolaryngol.

[44] Taklalsingh N, Falcone F, Velayudhan V (2017). Gradenigo’s syndrome in a patient with chronic suppurative otitis media, petrous apicitis, and meningitis. Am J Case Rep.

[45] Mani S, Rekha A (2017). Unusual case--revisited--Gradenigo’s syndrome. J Evol Med Dent Sci.

[46] Kazemi T (2017). Acute otitis media-induced gradenigo syndrome, a dramatic response to intravenous antibiotic. Iran J Otorhinolaryngol.

[47] Al-Juboori A, Al Hail AN (2018). Gradenigo's syndrome and labyrinthitis: conservative versus surgical treatment. Case Rep Otolaryngol.

[48] Brunet-Garcia A, Barrios-Crispi MV, Faubel-Serra M (2018). Carotid canal bone erosion. Gradenigo's syndrome. Acta Otorrinolaringol Esp (Engl Ed).

[49] Aksoy A, Varoglu AO (2018). Recurrent petrositis due to nasopharyngeal carcinoma in an adult patient: Gradenigo's syndrome. J Cancer Res Ther.

[50] ThakurR. ThakurR. (2018). A case of abducens nerve palsy: Gradenigo’s syndrome. Published online.

[51] Petrenko O, Talal A, Belissa R, Wiese-Rometsch W (2019). Invasive rhinocerebral mucormycosis leading to Gradenigo’s syndrome in type I diabetic. Endocr Pract.

[52] Bowman C, Nakhla N, Amedu V (2020). A rare complication of otitis media: Gradenigo’s syndrome successfully managed on outpatient antimicrobial therapy. Clin Infect Pract.

[53] Esmanhotto BB, de Araújo G, da Silva Faco A Jr, Pasqualli A, Yared JH (2020). Gradenigo's syndrome in a woman with systemic lupus erythematosus. Acta Neurol Belg.

[54] Savasta S, Canzi P, Aprile F, Michev A, Foiadelli T, Manfrin M, Benazzo M (2019). Gradenigo's syndrome with abscess of the petrous apex in pediatric patients: what is the best treatment?. Childs Nerv Syst.

[55] Rossi N, Swonke ML, Reichert L, Young D (2019). Gradenigo's syndrome in a four-year-old patient: a rare diagnosis in the modern antibiotic era. J Laryngol Otol.

[56] Chandran A, Sagar P, Monga R, Singh S (2020). Unusual manifestation of Koch's disease: Gradenigo-Lannois syndrome. BMJ Case Rep.

[57] Guimaraes GC, de Freitas PP, da Silva VA, Castilho AM (2021). Conservative management of petrous apex abscess and Gradenigo's syndrome in a diabetic patient: case report and literature review. Clin Case Rep.

[58] Hodges J, Matsumoto J, Jaeger N, Wispelwey B (2020). Gradenigo's syndrome and bacterial meningitis in a patient with a petrous apex cholesterol granuloma. Case Rep Infect Dis.

[59] McLaren J, Cohen MS, El Saleeby CM (2020). How well do we know Gradenigo? A comprehensive literature review and proposal for novel diagnostic categories of Gradenigo's syndrome. Int J Pediatr Otorhinolaryngol.

[60] Kale MV, Gaikwad NS, Chhabria SC (2020). Gradenigo’s syndrome: a petrous apex lesion. Int J Otorhinolaryngol Head Neck Surg.

[61] Nilam US, Dharmishtha RK, Anjali T (2020). A rare presentation of Gradenigo’s syndrome. J Otolaryngol Rhinol.

[62] Ghammam M, Gdissa A, Bellakhdher M (2020). Gradenigo’s syndrome a rare complication of acute otitis media: case report and literature revue. Egypt J Ear, Nose, Throat Allied Sci.

[63] Parekh MA, Pacheco VH (2021). Gradenigo's and Vernet's syndrome in an adult man with Candida mastoiditis. BMJ Case Rep.

[64] Anghel I, Anghel AG, Soreanu CC, Dumitru M (2012). Craniofacial trauma produced by a violent mechanism Coltea ENT Clinic experience. Rom J Leg Med.

[65] Vrinceanu D, Berghi ON, Cergan R, Dumitru M, Ciuluvica RC, Giurcaneanu C, Neagos A (2021). Urban allergy review: allergic rhinitis and asthma with plane tree sensitization (Review). Exp Ther Med.

